# Supplementary material for: Can increased prenatal exposure to thyroid hormones alter physiology and behaviour in the long-term? Insights from an experimental study in Japanese quails
Source: PeerJ. 2026 Jan 22;14:e20664. doi: 10.7717/peerj.20664 (PMC12832058; doi:10.7717/peerj.20664)

Tonic immobility

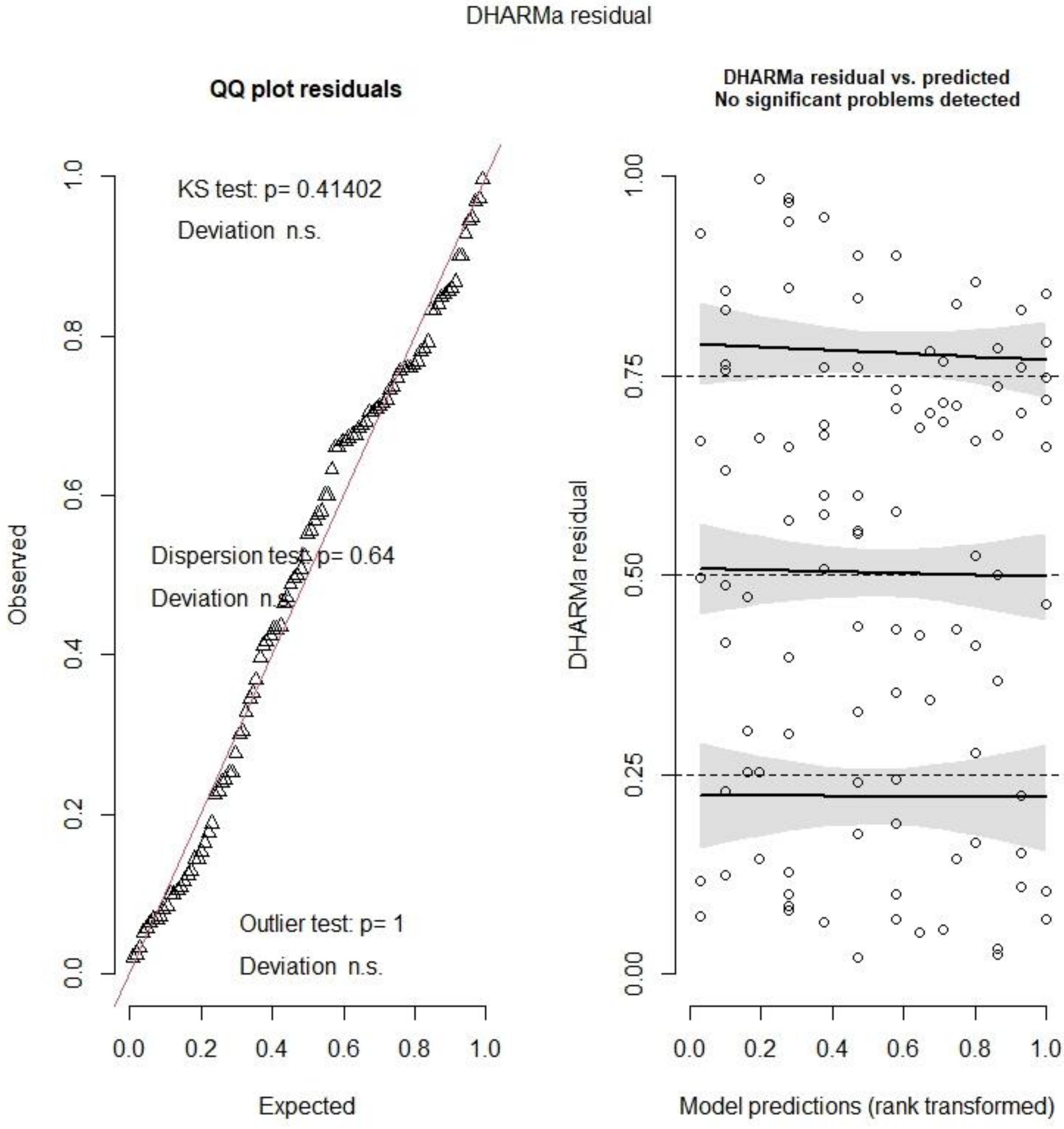

Open arena, chick escapes

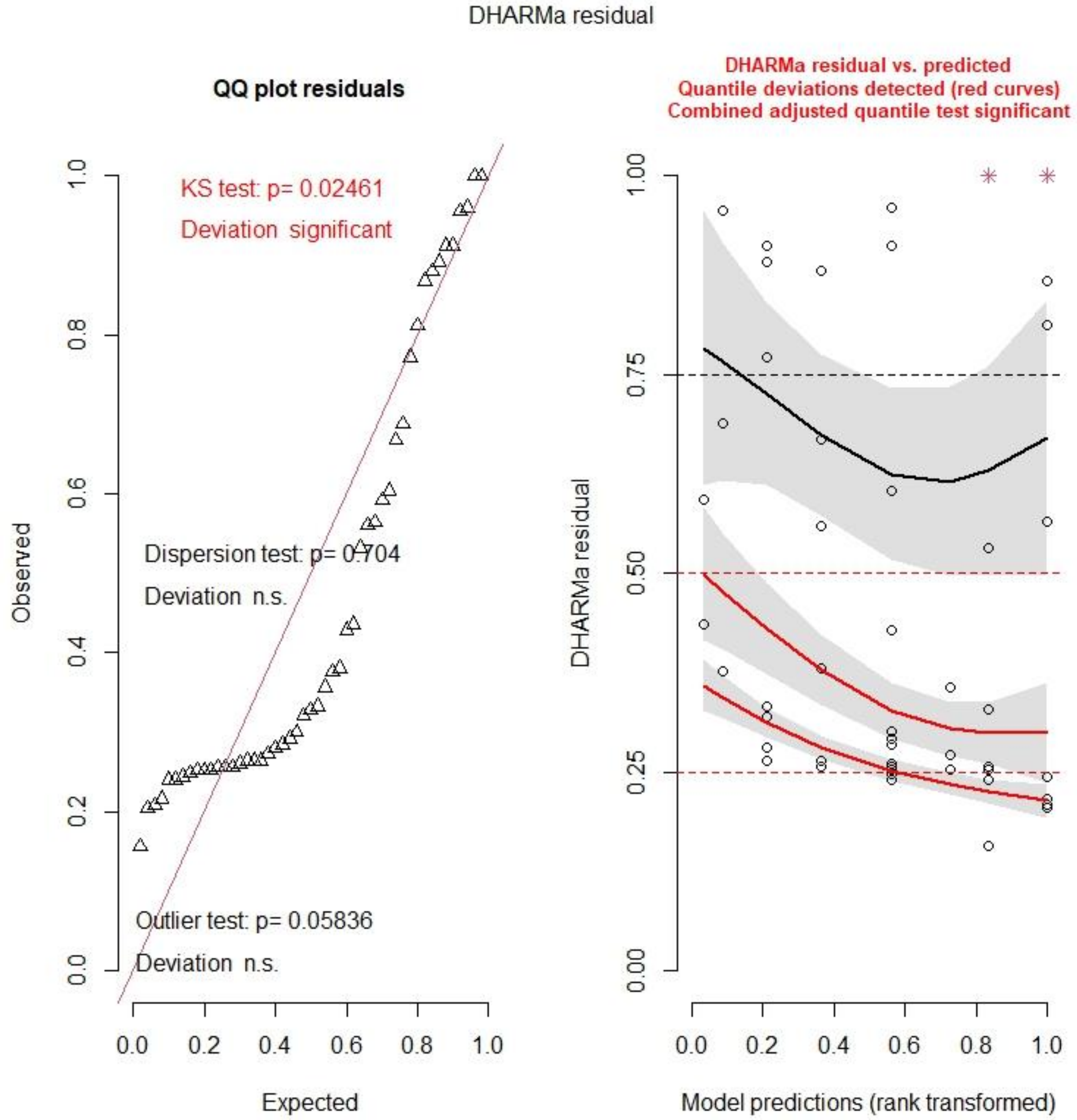

Open arena, movement duration

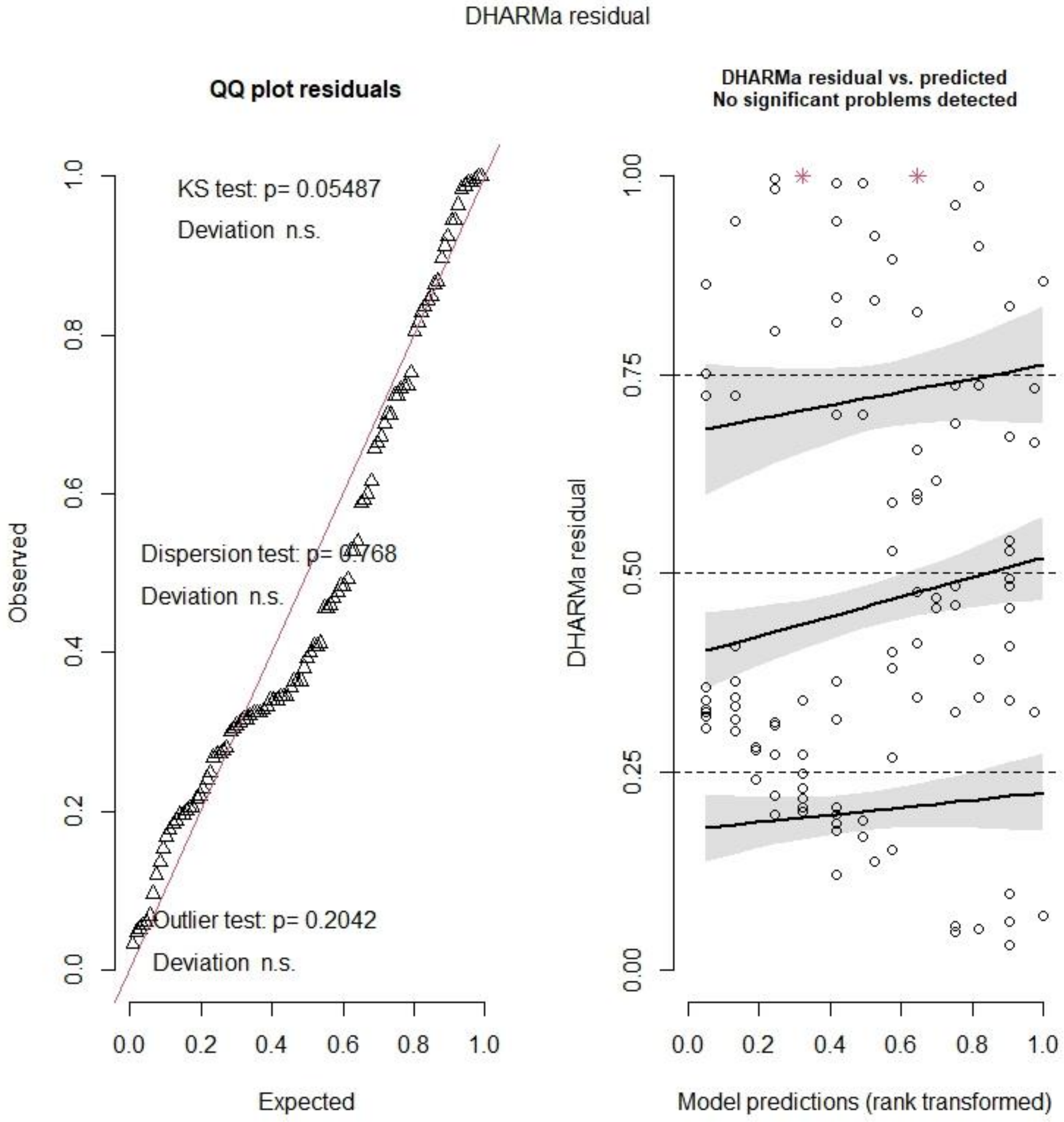

Emergence test, time until exit

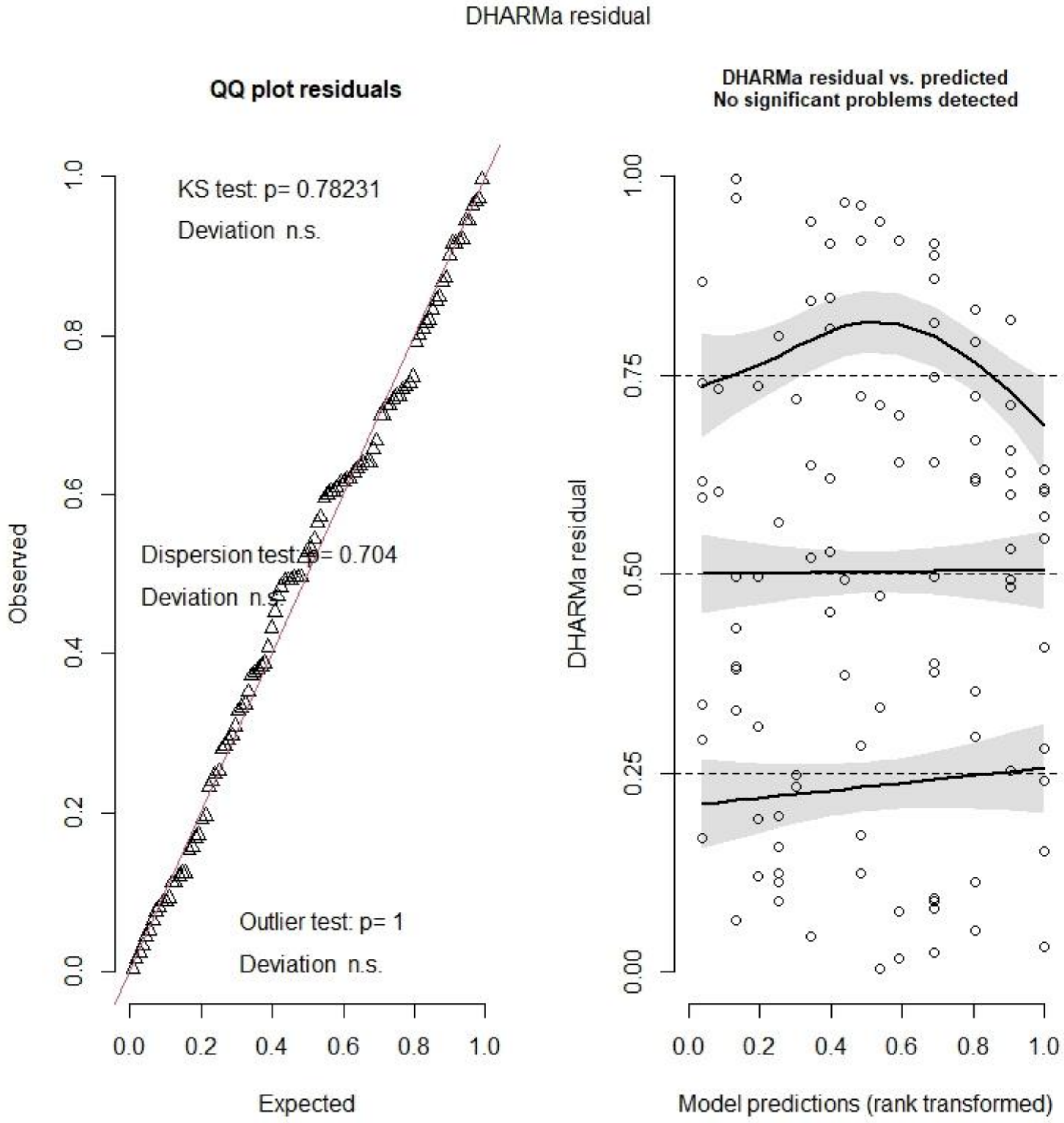

Emergence test, likelihood of emergence

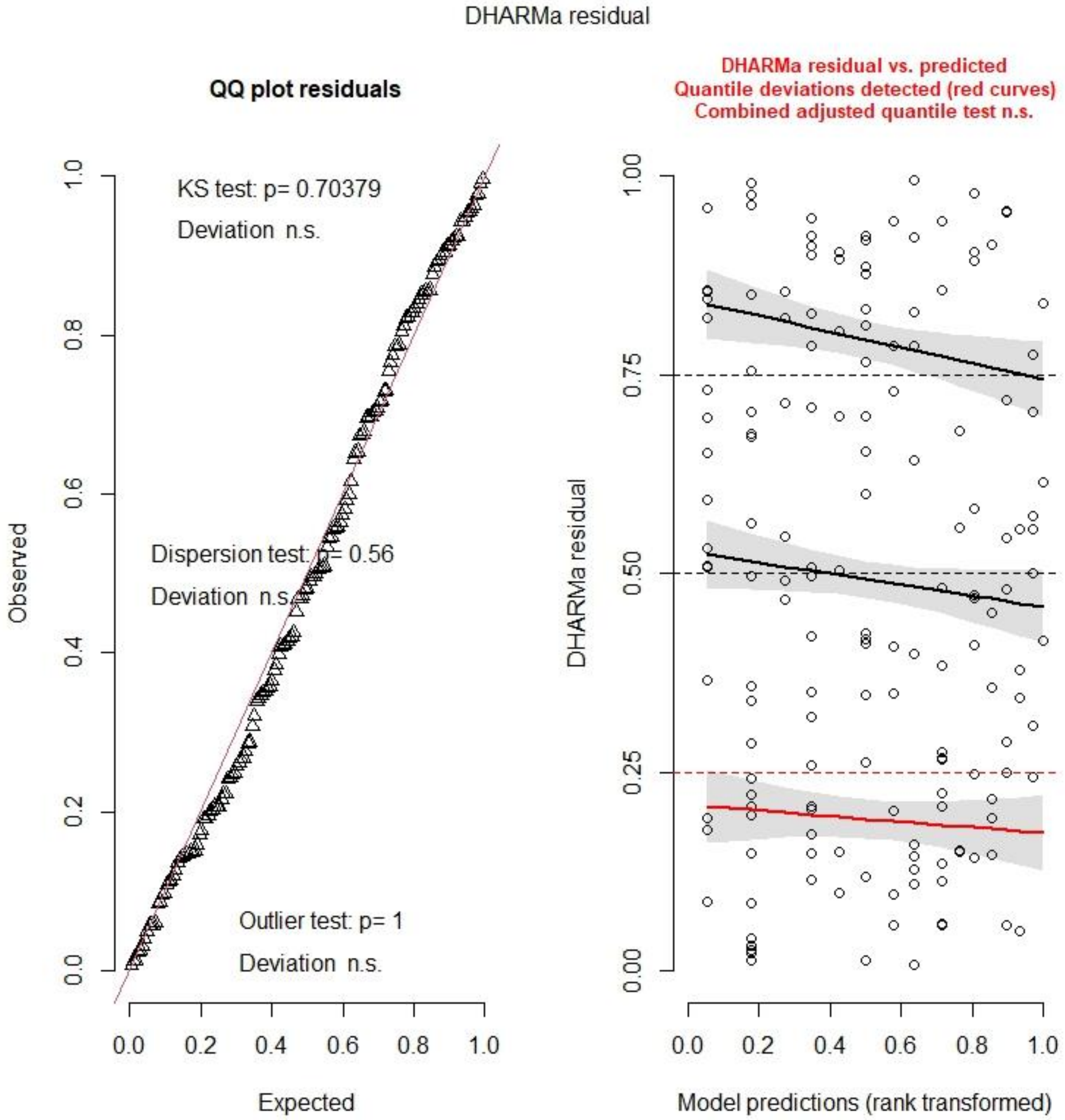

T3 concentration

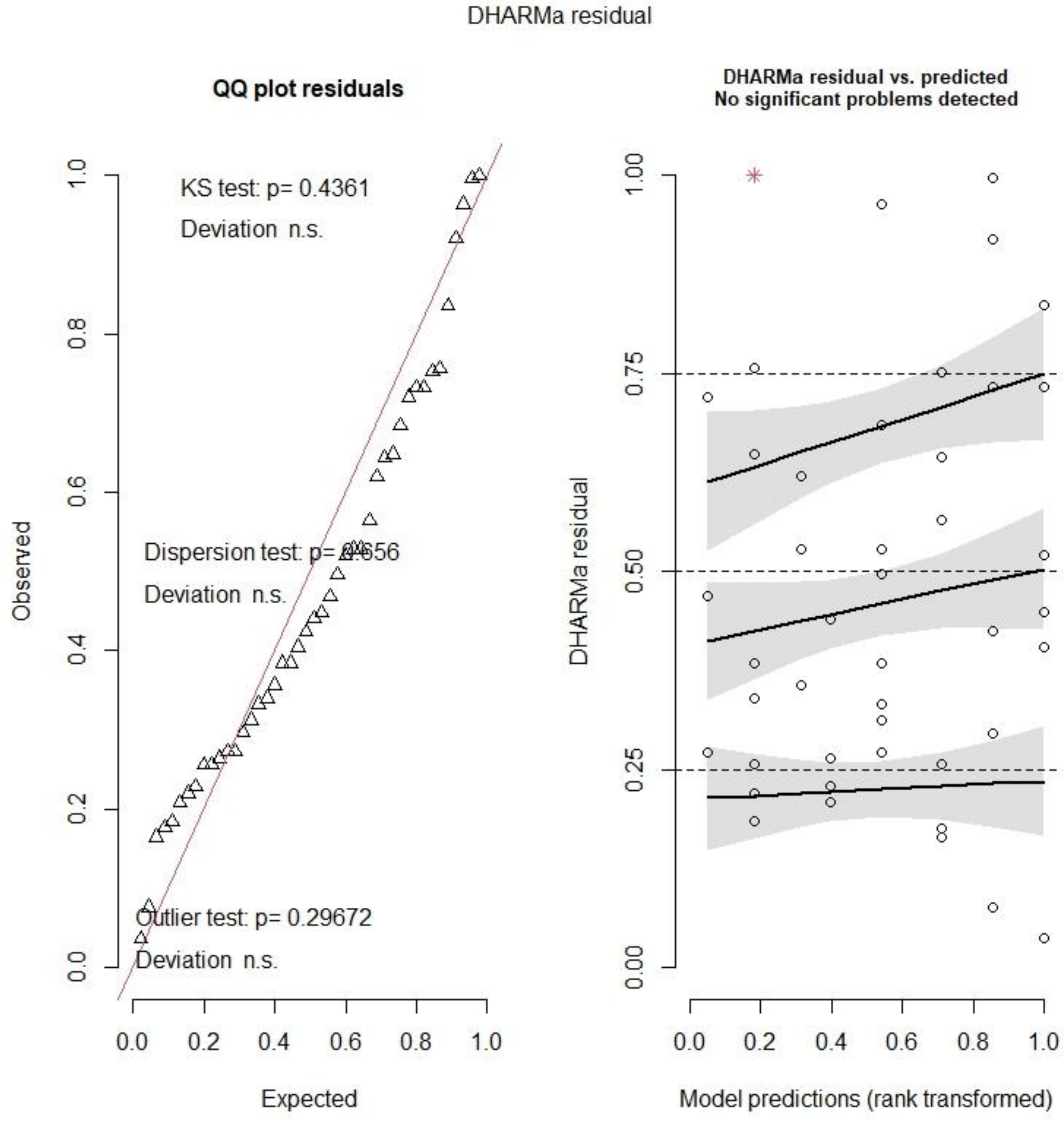

T4 concentration

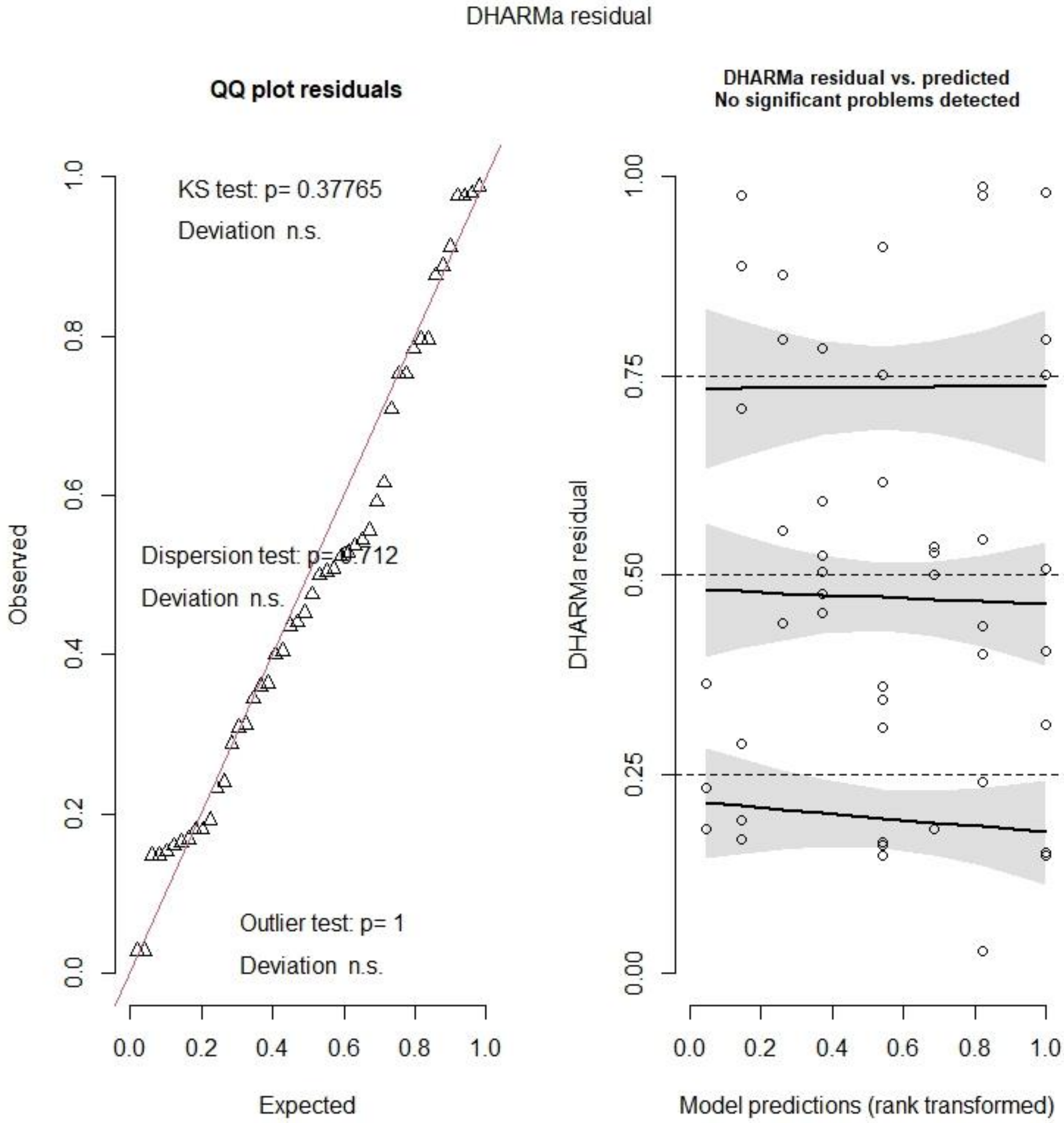

THRA expression

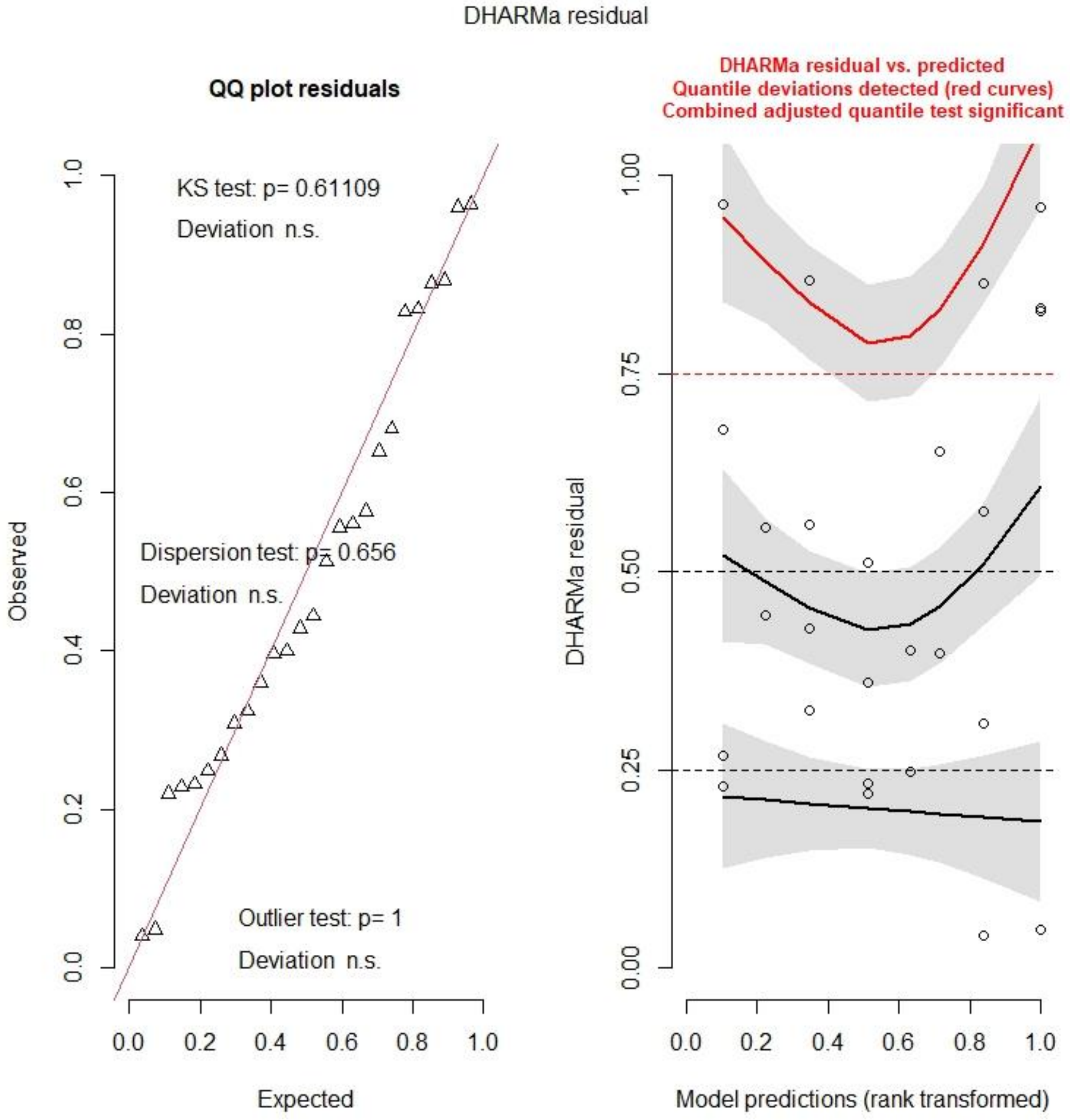

NCOA1 expression

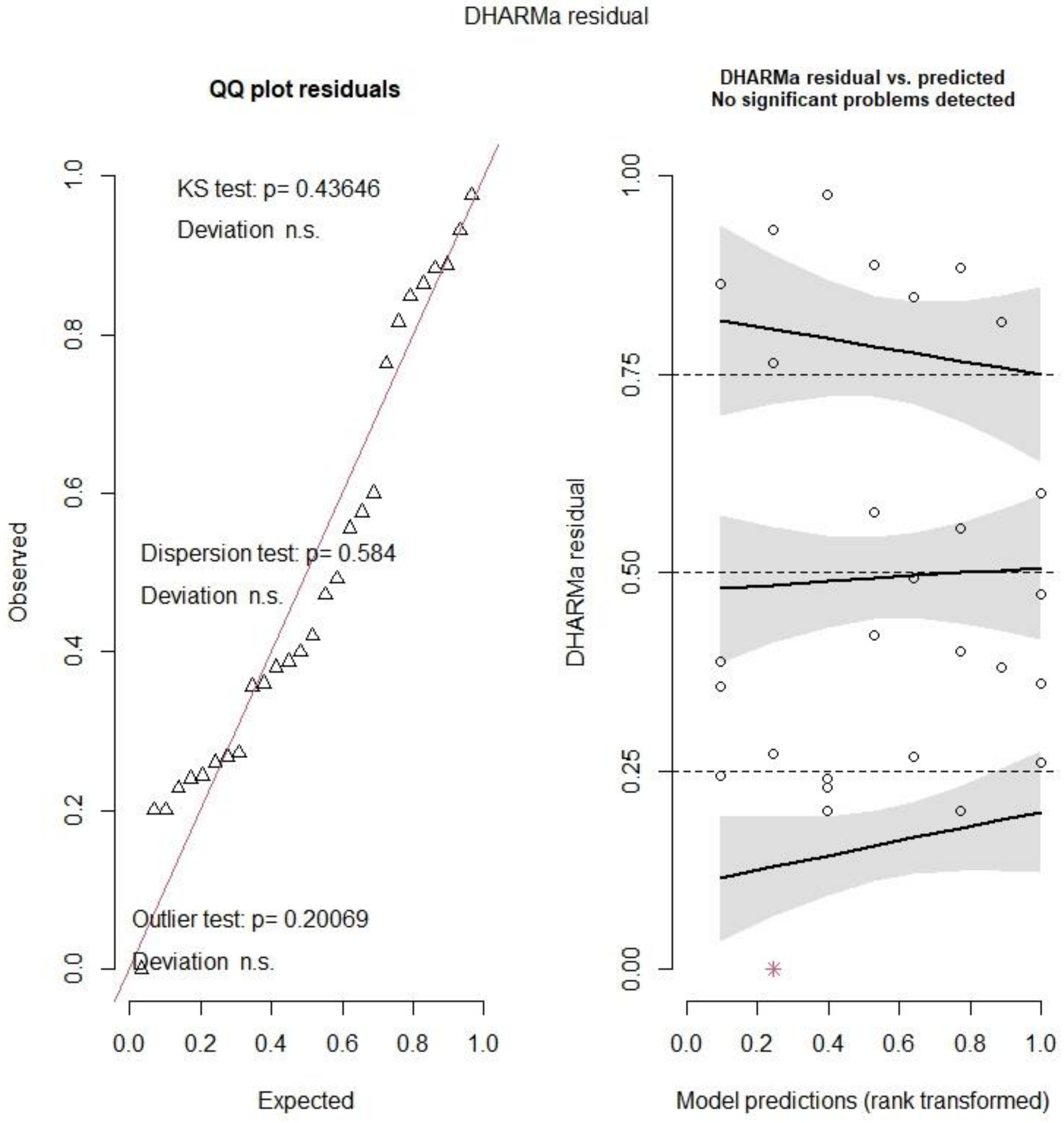

DIO2 expression

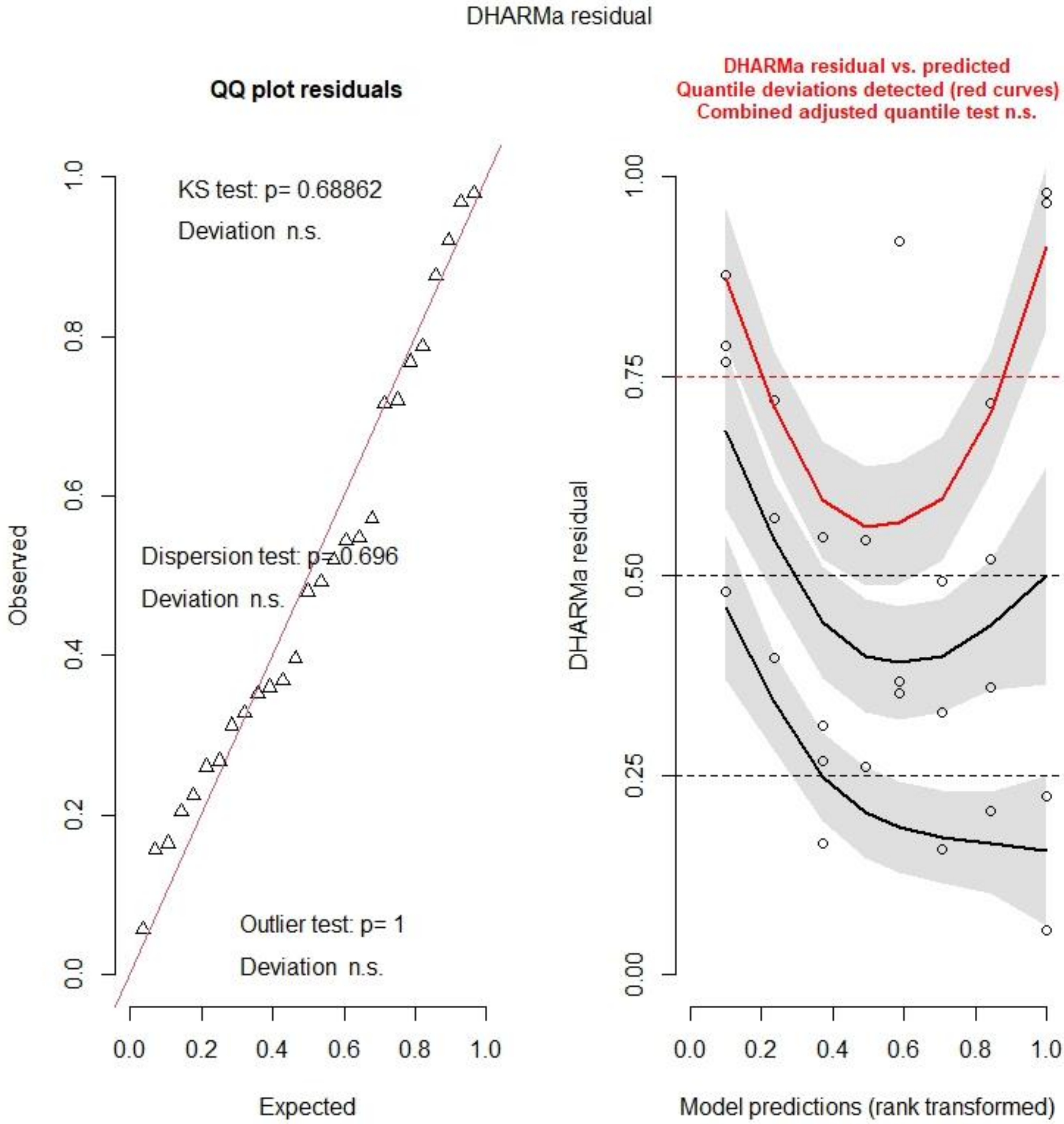

Supplement: Supplemental Information 4 — Quantile-quantile and residuals against fitted values plots, and outlier tests. Done using DHARMa package in R. [file peerj-14-20664-s004.pdf]
